# Domesticated chickens interact more with humans and are more explorative than Red Junglefowl

**DOI:** 10.3389/fvets.2024.1523047

**Published:** 2025-01-07

**Authors:** Ruth Demree, Per Jensen

**Affiliations:** ^1^Department of Animal Science, University of California, Davis, Davis, CA, United States; ^2^AVIAN Behavioural Genomics and Physiology Group, Department of Physics, Chemistry and Biology (IFM), Linköping University, Linköping, Sweden

**Keywords:** activity, chicken, contact-seeking, domestication, familiarization, human-animal relationship

## Abstract

Domesticated species are adapted to thrive in an environment with regular human interaction, and these interactions influence the development of a human-animal relationship. Chickens are the most abundant domesticated species, but their relationship with humans is poorly understood. A more comprehensive analysis of this relationship would provide valuable insight into their welfare needs. The present study compares the behavior of a domesticated and a non-domesticated breed of *Gallus gallus* in the presence of a familiar human. The domesticated breed was more active overall, and displayed more human contact-seeking behavior. These behavioral differences contribute to our understanding of the human-chicken relationship and could be helpful new insights for improving welfare of chickens in agricultural practice.

## 1 Introduction

Domestication is characterized by adaptations that help species thrive in a captive human environment. These adaptations can be physiological [e.g., changes in body size, egg size, and feed efficiency (1–3); changes in brain size and composition (4)] and behavioral [e.g., reduced fear response and increased stress tolerance (5); changes in intraspecific social behavior (6)], comprising an animal that is distinct from its wild ancestor. The most abundant domesticated animal on the planet is the chicken [*Gallus gallus domesticus*; (7)], and chickens differ greatly in appearance and to some extent in behavior from their wild ancestor, the Red Junglefowl (*Gallus gallus*). Chickens are not only the most numerous species in the global food production, but also have an increasing value as emotional support animals and in hobby breeding (7). When compared to Red Junglefowl, domesticated chickens are larger, lay more and larger eggs, react less strongly to acute restraint stress (8), and have reduced fear responses in general (9).

Fear of humans is a significant welfare concern because the captive environment necessitates regular human-animal interaction (10). The study of these interactions has developed independently in different animal groups (e.g., in the study of companion animals vs. research animals). Due to the resulting conflicts in methodology and terminology, it is arguably not a unified field (11). Within the study of agricultural animals, however, this field of research commonly centers around what is referred to as the human-animal relationship (hereafter HAR). The HAR is built upon previous interactions between a human and animal which then influence future interactions (12). This relationship is expressed through mutual behaviors, and can either be positive (e.g., signified by friendly and trustful interactions), negative (e.g., associated with punishment), or neutral (e.g., without any direct interactions). In chickens, a negative HAR can lead to increased instances of panic or violent escape attempts, harming both poultry production and welfare (13).

Generally, HAR research has centered around sociocognitive abilities in relation to humans: the ability of an animal to distinguish between individual humans, interpret human behavior, and communicate with and learn socially from humans (14). Human contact-seeking behavior, in which an animal approaches and looks at a human, has been described in many species, including dogs, horses, and goats (15–18). In a study comparing “looking behavior” between socialized dogs and wolves, the dogs would look to a human when faced with an impossible task, while the wolves would not (15), implying that human contact-seeking is a behavioral change that arose through the domestication process. Stress in dogs can also be reduced through periods of human contact (19). This demonstrates that social buffering, the phenomenon through which stress is reduced by the presence of a familiar companion, can occur between an animal and a human (20–22).

The human-chicken relationship has received relatively little scientific attention, despite the direct impact of humans on chicken welfare. Most research has focused on how chicken welfare is affected by environmental factors, such as cage size and equipment, group size, etc. However, the two most common stressors experienced by captive chickens are unexpected changes to the physical or social environment, and human exposure (23), and improvements of HAR can therefore potentially be an important source of increased welfare. Direct contact between a chicken and a human has been shown to establish predatory overtones in an Open Field (24), and chickens will remain in tonic immobility for longer durations when a human is staring directly at them (25). However, familiarization through periods of gentle contact has been found to reduce fear responses in adult laying hens (26, 27). This has also been found in chicks of three different domesticated breeds, although the experimenters acknowledged breed-level differences (28).

The present study aims to further describe the human-chicken relationship by investigating domestication effects on *inter*specific sociocognitive ability. Like dogs, domesticated chickens are descended from a group-living, highly social species with demonstrated *intra*specific sociocognitive ability. It is expected that, when compared to the Red Junglefowl, the domesticated chicken will interact more with a human, and will be less fearful when a familiar human is present. These behavioral differences will contribute to our understanding of the human-chicken relationship and how this may have been evolving as a result of domestication, and could gain helpful new insights for improving welfare of chickens in agricultural practice.

## 2 Materials and methods

### 2.1 Animals and housing

The experiments were approved by the local ethical committee for animal experimentation in Linköping, license number 14916-2018. All of the birds used in the study are from breeding populations that are housed within the chicken facilities of Linköping University and kept under identical rearing conditions. They were transferred from the chick rearing facility to the pullet facility at 5 weeks of age. The experiment started when the birds were 42 weeks of age and lasted for 7 weeks. The birds consisted of two breed groups: female White Leghorn laying hybrid (SLU 13, hereafter WL, *N* = 14) originating from a Scandinavian selection and crossbreeding experiment (29), and female Red Junglefowl (S12, hereafter RJF, *N* = 16) the twelfth captive-bred generation originating from the grandchildren of a population that had been brought to Sweden from Thailand and bred in a zoo (30). The home pens of each breed group consisted of an indoor enclosure (ground floor = 2.50 × 3.00 m, ceiling height = 2.90 m) and a connected outdoor enclosure (floor = 2.50 × 4.05 m, ceiling height = 2.50–2.90 m). At the ages of this study, bird weights are typically 900 g (RJF), and 1,300 g (WL).

At the beginning of the test week, birds were transported (carried in a standard transport cage) from the home pen to test pens in an adjacent lab room ~10 m down the hall, where they were housed in pairs of the same breed. The ordering of the test pairs was counterbalanced across the entire testing period. Four test pens were constructed within the lab room with temperatures maintained between 15 and 20°C and a relative humidity of 40%−70%, and natural light was let in through the windows to reflect the current day/night cycle. Each pen measured 1.20 m × 1.20 m (1.80 m in height), with netting secured over the top to stop birds from flying out of the pens, and a plexiglass and cardboard barrier over the walls to inhibit vision into other pens. The pairs were provided with a cylindrical wooden perch (~0.85 m long) and a layer of wood shavings on the floor, and provided access to feed, water, and oyster shell (in a separate feeder) *ad libitum*. The supply of feed and oyster shell was maintained by a technician; the birds did not see the experimenter handle feed at any point. The pairs were always tested together, except in the Open Field test (see below) where there were tested individually.

All tests were carried out by one and the same experimenter (the first author), who has extensive previous training on recording chicken behavior. The experimenter was continuously supervised by the second author and other experienced members of the lab.

### 2.2 Open field arena

An arena was constructed in a separate lab room for the Open Field trials. The arena measured 2.04 m × 2.40 m (1.80 m in height) with netting secured over the top, and it was virtually divided into a 6 × 6 grid, each square measuring 0.40 m × 0.40 m. A piece of cardboard was placed over one wall as a visual barrier for the experimenter to stand behind during trials without human presence. The floor of the arena was covered in a layer of wood shavings. An aluminum soda can (either green or yellow) was placed inside of the arena to function as a novel object, and lamps were set up within the test room so that each trial could begin in complete darkness. The color of the novel object within the arena was swapped in between trials. Which novel object was used in which trial was counterbalanced across groups.

### 2.3 Experimental design

Data collection began on 12th September 2022 and concluded on 28th October 2022, lasting for a total of 7 weeks. Each week followed the same 4-day testing schedule with a new test group. One trained experimenter conducted all of the trials and wore the same pair of blue overalls during all trials. After familiarization, the birds were exposed to two different tests assessing human contact-seeking and fearfulness in a novel environment, both examples of practically relevant events that could be associated with the quality of the HAR.

#### 2.3.1 Familiarization

For the first 3 days, the experimenter sat inside each test pen for 15 min. The experimenter spoke quietly to the test pair throughout the procedure, in a calm tone characterized by longer duration sounds (31). During this phase the birds were within 2 m of the experimenter, who assumed a neutral face expression throughout the procedure. This was repeated in each test pen.

#### 2.3.2 Human interaction

Birds were tested three times in pairs over the first three trial days. Following each familiarization session, the experimenter reentered the test pen and knelt, holding out a small empty bowl just above the eye level of the test pair, ensuring that they could not see inside. Each session began with a verbal cue, then the experimenter held out the bowl with a neutral expression for 30 s. The reason for holding out the bowl was to have a single focal point toward which the bird's head and body orientation could be measured. Trials were recorded on a GoPro Hero 11. Each test pair was scored together for the total time spent with both their head and body facing the bowl in the experimenter's hands.

#### 2.3.3 Open field

Birds were tested twice on the 4th day of the week. An individual bird was transported to the arena by the experimenter and placed on the ground in darkness. The trial began when the lamps were switched on, and the bird was able to explore by walking around the arena for 5 min. Each individual bird was tested twice (with at least 1 h between trials; the birds were returned to their familiarization pens inbetween the trials), once alone and once with the experimenter seated quietly in the corner of the arena. The order of the trials was counterbalanced, and the corner that the experimenter sat in was the same across all trials. Trials were recorded, and a virtual 6 × 6 grid was overlaid on the arena on the video images (so there were no grids or line in the physical arena). The individual bird's overall activity was scored as the total number of times that it crossed a grid line. No further behavioral recordings were done.

### 2.4 Data analysis

The data were analyzed using Generalized Linear Models in SPSS software (29.0.2.0). A “normal” probability distribution and link function “identity” were used to assess the behavior of each breed. The Human Interaction model included breed (WL vs. RJF) and day. The Open Field model included breed and human presence (Human vs. No Human).

## 3 Results

Summary measures for each breed are listed in Table 1. Five trials were removed from the analysis for Human Interaction due to technical issues with the video recording (3/24 RJF, 2/21 WL).

**Table 1:** Data collected in both the human interaction test and the Open Field test.

**Trial**	**Behavior**	**Breed**	**Count**	**Mean**	**SD**	**Median**	**Minimum**	**Maximum**
Human interaction	Head/body facing bowl (seconds)	RJF	21	1.9	3.97	0	0	12
		WL	19	11.7	8.35	12	0	27
Open field	Activity (crossed a gridline)							
	Human	RJF	16	39.1	39.80	24.5	3	126
		WL	14	85.4	45.70	80.5	11	176
	No human	RJF	16	53.1	42.37	42.0	7	144
		WL	14	86.1	46.58	80.5	33	174

The table shows the behavior variables in each of the tests, the breed being tested, as well as the number of animals tested in each. Furthermore, the table gives the mean, standard deviation, median, minimum, and maximum of all data.

### 3.1 Human interaction

**Figure 1:**
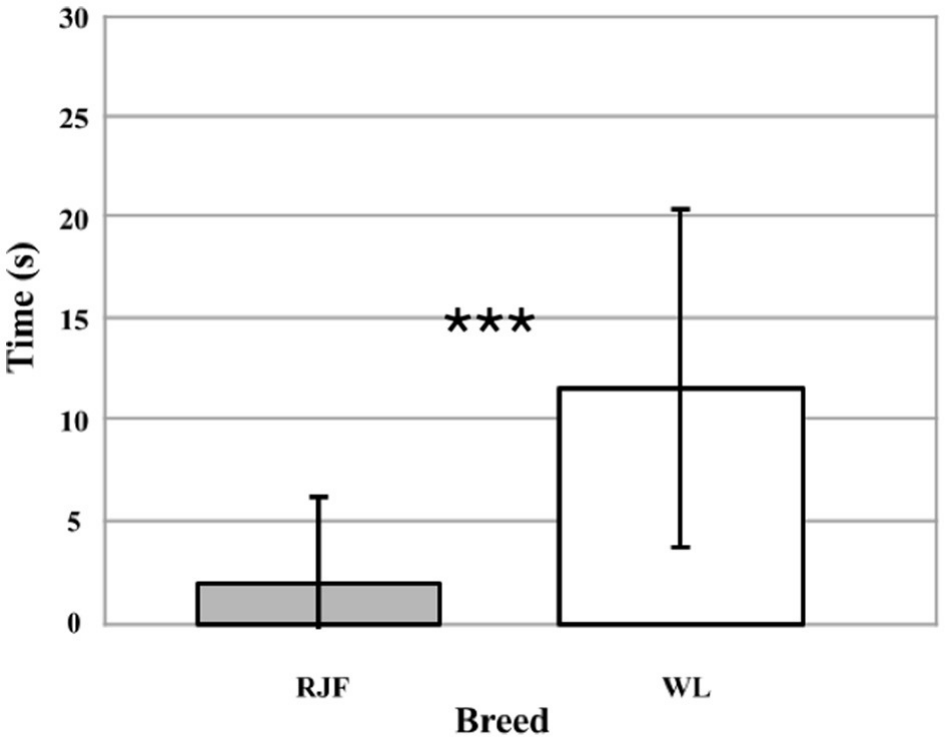
Time (seconds) spent facing the bowl in the experimenter's hands for each 30-s trial (*N* = 40). Comparing between White Leghorns (WL) and Red Junglefowl (RJF). Error bars represent the standard deviation (SD). Due to the lack of effect of trial day (*p* = 0.732), data points from all 3 days have been combined. The asterisks indicate a significant effect of breed (*p* < 0.001) on time spent facing the bowl.

During the Human Interaction trials, the birds were tested three times in pairs (*N* = 40). The trial day (1, 2, or 3) did not affect how much time the test pairs spent facing the bowl in the experimenter's hands (*p* = 0.732). Breed did have a significant effect (Wald χ^2^ = 47.265, df = 1, *p* < 0.001). The RJF pairs interacted with the experimenter significantly less than the WL pairs (Wald χ^2^ = 24.896, df = 1, *p* < 0.001; Figure 1). In spite of relative large variation within breeds, on average the WL spent more than five times as much time interacting with the human as the RJF.

### 3.2 Field

**Figure 2:**
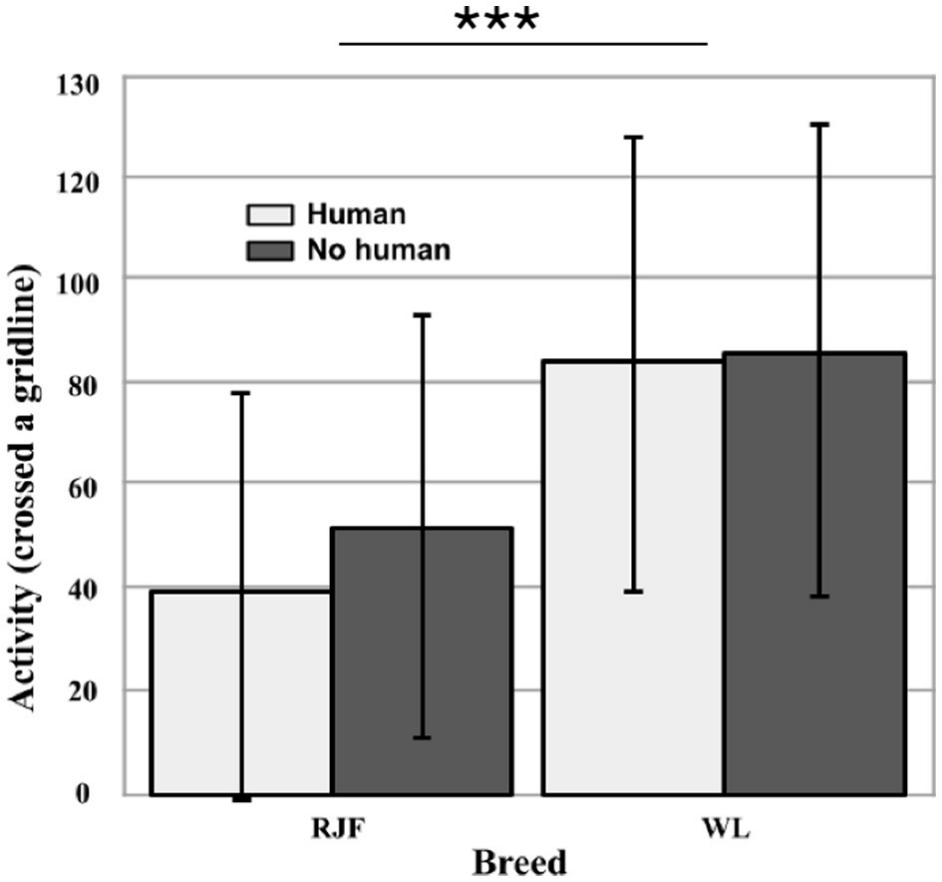
Activity (total number of times the individual crossed a gridline) in an Open Field, with and without human presence (*N* = 60). Comparing between White Leghorns (WL) and Red Junglefowl (RJF). Error bars represent the standard deviation (SD). There was a significant effect of breed (*p* < 0.001) on activity (indicated by asterisks), however human presence had no effect (*p* = 0.474).

During the Open Field trials, each bird was tested twice individually, one without human presence and one with the experimenter seated within the arena (*N* = 60). Breed had a significant effect on activity in the arena (Wald χ^2^ = 71.859, df = 1, *p* < 0.001). The RJF moved through the arena less than the WL (Wald χ^2^ = 13.203, df = 1, *p* < 0.001; Figure 2). This was found to be consistent between trials: human presence did not have a significant effect on activity (*p* = 0.474). Again, there was a relatively large within-breed variation, but still the WL on average were about twice as active as the RJF measured by the number of gridline-crossings.

## 4 Discussion

Here we show breed-level differences in chicken behavior to human presence. The White Leghorn established a greater degree of visual contact with the experimenter, orienting its head and body toward the bowl in the experimenter's hands for significantly longer than the Red Junglefowl. The White Leghorn was also more active in the Open Field, regardless of whether or not the experimenter was present.

Both Red Junglefowl (32) and domesticated chickens (33) can derive information from following a conspecific's gaze. However, White Leghorns have been shown to be more successful at social learning through observation than Red Junglefowl (34). How this translates to the human-chicken relationship has not been previously studied. The present findings on human contact-seeking reflect the results when comparing dogs and wolves (15), further implying that this behavior is strengthened by the domestication process. Whether or not chickens can learn socially by observing humans is still unclear. Previous study on avian species has shown that social learning from humans is context-dependent: for example, although socialized ravens can follow the gaze of a human experimenter (35), they cannot locate hidden food based on human gaze cues alone (36). To our knowledge, the present study is the first to specifically assess HAR as a function of human interactions, and we are not aware of any other studies that specifically have measured, for example, duration of human contacts or activity levels in an Open Field with and without human presence.

In the present experiment we specifically aimed at assessing the reactions of the birds in two specific situations, previously not studied in chickens. The first was to measure their propensity for human contact-seeking, and the other to study their reactions to changes in their physical and social environment and how this was affected by human presence. Both these tests were deemed essential for assessment of HAR, a previously overlooked aspect of chicken behavior. There are of course many other tests that could have been included to assess, for example, fearfulness, but in this study we attempted to focus mainly on the HAR.

The Open Field trials aimed to investigate the impact of human presence in a stressful situation. A visible human in an Open Field increases ambulation latency in chicks reared with minimal exposure to humans (37), but familiarization has been shown to lower their fear response to human presence (26). In the present study, activity remained consistent regardless of human presence, which indicates that the familiarization process successfully habituated the birds to the experimenter. However, the Red Junglefowl were less active overall, and had a numeric—but not significant—tendency to be less active when a human was present. Domestication favors individuals that show less fear and aggression toward humans (38), and accordingly, Red Junglefowl have been found to exhibit a stronger general fear response than White Leghorns (9), which may include reduced activity in an Open Field. It is important to note that Red Junglefowl are much smaller than White Leghorns (fully grown WL hens are over 2.0 kg, whereas RJF hens are around 1.0 kg), and activity in the Open Field was measured using gridlines of a standard length. The White Leghorn group may have appeared to be more active because of a difference in stride length. However, White Leghorns move more through an Open Field than either the Green-legged Partridge or the Polbar, two other layer breeds of similar size (39). In light of these intergroup differences, activity itself may be an insufficient proxy for stress in an Open Field. Future studies should therefore include detailed behavioral measures such as head-flicks, vigilance and flight attempts.

To our knowledge, there has been no previous investigation into whether human presence could possibly buffer stress in chickens. Interspecific social buffering has been previously demonstrated between humans and dogs (19), and more recently, between humans and goats (40). Hens and chicks show both the arousal and buffering of stress when observing one another (41, 42), and so it is possible that human presence could act as a social buffer through the development of a positive HAR. However, in the present study there was no significant effect of human presence on activity. Previous studies have demonstrated that positive physical contact, such as gentle handling and petting, also helps habituate chickens to human presence and reduces overall fear of humans (26, 27, 43). In the present study, familiarization consisted of several sessions of calm and positive contact, both visual and verbal, but not physical (a handful of individuals from both breed groups did establish physical contact by perching on and investigating the experimenter, but this was not initiated by the experimenter). However, it was necessary to handle the birds in this study when transporting them between the test pen and the arena, which may have added a predatory overtone to the Open Field trials with human presence (24). In a future study, positive physical contact should be incorporated into the familiarization process to see whether this could further improve the HAR. The present study was limited by the sample size of the breed groups, and future studies comparing behavior between a broader representative sample will be necessary to apply these findings to the human-chicken relationship in general, its relationship to domestication effects, and its applications to poultry welfare. Moreover, the effect of age of the chickens on HAR should be investigated, since here we only studied adult animals (albeit with similar rearing conditions and prior experiences). In practice, experiences of human contact may vary extensively between different populations, for example as a result of handling in the hatcheries. To make the experiment more relevant for practical situations, we have only studied females, so a further development would be to include the effects of presence of a male in a female group on the development of HAR.

We did not observe any reduction in contact-seeking over the test days in any of the breeds, which indicates that the birds did not habituate to the test situation, as would have been expected if the human presence would have been perceived as a stressor. This appears to validate that the behavior we observed was a human-animal interaction based on a preexisting relationship rather that a response to a perceived stressful stimulus.

Chickens are the most abundant domesticated animal in the world, with a global population of over 33 billion individuals as of 2020 (7), and yet the human-chicken relationship has received very little scientific attention. In addition, chicken research is often generalized across breeds, even though chicken breeds vary in their behavioral repertoires (39). One could blame our inherent mammalian bias, or perhaps it is because of the bird's great abundance: they are so internationally ordinary that fewer people are attracted to study them. Regardless, a more conscious effort needs to be made in future research to give these birds proper attention. They are a highly social animal with demonstrated sociocognitive abilities, and studies that describe these birds as such help to foster healthy human-chicken relationships and ultimately improve chicken welfare.

An important caveat to the present study is of course the selection of the specific bird strains used. The RJF had been kept in captivity for several generations, and therefore may have grown accustomed to human presence. We cannot exclude that the results would be even clearer if we had used wild-caught birds for comparison. However, during the time the birds have been kept in the lab, great care has been taken to treat them in the same way as other birds in the facility, which means minimal human interactions. The fact that there were large differences in the behavior of the two strains is therefore a strong indication that these birds still have retained much of their natural shyness, and the results are therefore totally compatible with the hypothesis that domestication has caused an inherent increase in human-directed interest and thereby facilitated the development of a more intense HAR. Furthermore, we have compared the RJF with a specific WL strain, and it cannot be excluded that the results would have been different with another strain. The WL used in this study were from a non-commercial line, and thus not bred for any specific production trait, such as egg production or growth. It was chosen to represent a generic domesticated bird, and the fact that we observed the reported differences is an indication that HAR has been facilitated by domestication as such, not specifically by the last century's intense breeding for production traits in chickens.

## 5 Conclusion

In conclusion, we found that domesticated White Leghorn chickens interact more with humans than Red Junglefowl by establishing visual contact in a situation where they can choose to do so. Furthermore, we found that White Leghorns are more explorative than Red Junglefowl in a potentially stressful Open Field test, regardless of whether a human is present or not. The results form a basis for improving the HAR in practical chicken production, which may potentially have benefits for chicken welfare. Future studies should aim at assessing such improvements in practical settings, for example by studying how stress related behavior in production units can be counteracted by familiarization with caretakers and systematic improvements of HAR in these conditions.

## Data Availability

The raw data supporting the conclusions of this article will be made available by the authors, without undue reservation.
